# SARS-CoV-2 infection as a potential risk factor for the development of cancer

**DOI:** 10.3389/fmolb.2023.1260776

**Published:** 2023-09-11

**Authors:** Natalia Ogarek, Paulina Oboza, Magdalena Olszanecka-Glinianowicz, Piotr Kocelak

**Affiliations:** ^1^ Pathophysiology Unit, Department of Pathophysiology, Faculty of Medical Sciences in Katowice, The Medical University of Silesia, Katowice, Poland; ^2^ Students’ Scientific Society at the Pathophysiology Unit, Department of Pathophysiology, Faculty of Medical Sciences in Katowice, The Medical University of Silesia, Katowice, Poland; ^3^ Health Promotion and Obesity Management Unit, Department of Pathophysiology, Faculty of Medical Sciences in Katowice, The Medical University of Silesia, Katowice, Poland

**Keywords:** cancer, excess death, long Covid, oncogenesis, SARS-CoV-2 infection

## Abstract

The COVID-19 pandemic has a significant impact on public health and the estimated number of excess deaths may be more than three times higher than documented in official statistics. Numerous studies have shown an increased risk of severe COVID-19 and death in patients with cancer. In addition, the role of SARS-CoV-2 as a potential risk factor for the development of cancer has been considered. Therefore, in this review, we summarise the available data on the potential effects of SARS-CoV-2 infection on oncogenesis, including but not limited to effects on host signal transduction pathways, immune surveillance, chronic inflammation, oxidative stress, cell cycle dysregulation, potential viral genome integration, epigenetic alterations and genetic mutations, oncolytic effects and reactivation of dormant cancer cells. We also investigated the potential long-term effects and impact of the antiviral therapy used in COVID-19 on cancer development and its progression.

## 1 Introduction

Coronavirus disease 2019 (COVID-19), is caused by the novel severe acute respiratory syndrome coronavirus 2 (SARS-CoV-2). The clinical spectrum of COVID-19 ranges from asymptomatic to severe respiratory failure and death (Parasher, 2021). SARS-CoV-2 is mainly spread by respiratory droplets during face-to-face contact, such as coughing, talking, and sneezing, by symptomatic, pre-symptomatic, and asymptomatic carriers and, to a lesser extent by contaminated surfaces (Wiersinga et al., 2020). In addition, the virus has been shown to significantly modulate the immune system and induce low-grade chronic inflammation (Marshall, 2020; Saini and Aneja, 2021). Thus, SARS-CoV-2 infection in the long term may contribute to the development of cancers. Currently, cancer is one of the most important worldwide public health problems. Just in the United States, from January 2022 the number of cancer survivors was estimated at 18.1 million (National Cancer Institute, 2022). Some data suggested the role of SARS-CoV-2 as a potential risk factor for cancer development. In this review, we summarize the available data on the potential impact of SARS-CoV-2 infection on cancer development, including effects on host signal transduction pathways, immune surveillance, chronic inflammation, oxidative stress, cell cycle dysregulation, and potential viral genome integration. Moreover, we analysed the potential impact of the antiviral therapy used in the treatment of COVID-19 on oncogenesis and cancer progression.

## 2 Mortality among patients infected with SARS-CoV-2

The emergence of a global COVID-19 pandemic was an unprecedented public health crisis. The pandemic affected numerous people in approximately 200 countries and territories (Zhang et al., 2020). The mean in-hospital mortality is estimated at around 15%–20% and among patients admitted to the Intensive Care Unit (ICU) at 40% (Wiersinga et al., 2020). Moreover, the study analysing data from 191 countries and territories, and 252 regional units for selected countries from 1 January 2020, to 31 December 2021, estimated the number of excess deaths due to SARS-CoV-2 infections was nearly 3.07 times (95% CI: 2.88–3.30) higher than documented by official statistics (18.2 million vs. 5.94 million) (COVID-19 Excess Mortality Collaborators, 2022). Different chronic diseases including obesity, diabetes, hypertension, cancer, and respiratory and cardiovascular diseases are risk factors for a severe course of SARS-CoV-2 infection, admission to the ICU, and mortality (Gallo Marin et al., 2021). Numerous studies analysed the association between SARS-CoV-2 infection and mortality among patients with cancer. Higher mortality due to COVID-19 among men with cancer was found in the Italian population (Montopoli et al., 2020). In addition, a systematic review based on combined studies showed a significantly higher rate of coexisting malignancies among patients with a severe or deadly course of COVID-19 (7.3%–20.3% vs. 1%–3.9% of malignancies in all SARS-CoV-2 infected patients). Moreover, patients with cancer infected with SARS-CoV-2 have a significantly higher mortality range than patients without cancer (11.4%–35.5% vs. 3.8%–8.5%) (Ali et al., 2022).

## 3 The impact of SARS-CoV-2 infection on cancer outcomes

SARS-CoV-2 affects the host’s immune response, which may influence cancer outcomes. Furthermore, the co-occurrence of SARS-CoV-2 infection and cancer immunotherapy, such as immune checkpoint inhibitors, may modify the course of COVID-19. Moreover, lymphopenia related to chemotherapy, radiotherapy, and steroid therapy as well as a high ratio of neutrophils to lymphocytes are poor predictors of cancer progression and the course of COVID-19 (Ali et al., 2022). In addition, inflammation related to SARS-CoV-2 infection can affect cancer cell proliferation and survival, as well as angiogenesis, and metastasis. The infiltration of immune cells related to almost all cancers is a source of secretion of inflammatory cytokines including IL-6 and tumour necrosis factor–alpha (TNF-alpha), which is an important link to cancer–inflammation interactions. The increased release of the same cytokines has been observed in SARS-CoV-2 infection. Moreover, elevated levels of cancer-promoting growth factors have been observed in patients with haematological malignancies up to 3 months after SARS-CoV-2 infection (De Winter et al., 2021).

Of interest, a study including 500,000 American adults with COVID-19 showed a 7.8% mortality rate among patients with recently treated cancer, 5.0% among patients with non-recently treated cancer and 1.6% among patients without cancer. Furthermore, the adjusted analysis revealed similar mortality due to COVID-19 among patients with non-recently treated cancer and patients without cancer (OR = 0.93; 95% CI: 0.84–1.02) (Chavez-MacGregor et al., 2022). Moreover, the meta-analysis of 29 studies including 21,257 patients with lung cancer and SARS-CoV-2 infection showed significantly higher mortality due to COVID-19 among these patients (HR = 2.00; 95% CI: 1.52–2.63, *p* < 0.01) or patients with other malignancies (HR = 1.91; 95% CI: 1.53–2.39, *p* < 0.01) than in patients without cancer (Oldani et al., 2022). In addition, the Italian study found a higher proportion of deaths among patients with than without cancer infected with SARS-CoV-2 (14.7% vs 4.5%; *p* < 0.0001) (Rugge et al., 2020). It has also been shown that SARS-CoV-2 infection caused treatment delay, permanent discontinuation of treatment, or failure to perform scheduled diagnostic procedures (Tagliamento et al., 2020).

Data describing the impact of asymptomatic or mild symptoms of SARS-CoV-2 infection on treatment outcomes in cancer patients is scarce. Case reports describing a patient with primary mediastinal B-cell lymphoma and a patient with metastatic sigmoid cancer showed that asymptomatic SARS-CoV-2 infection did not contraindicate the use and continuation of chemotherapy (Woźniak et al., 2021). Similarly, Hempel et al. (2020) showed that asymptomatic SARS-CoV-2 infection did not affect the effects of chemotherapy. However, data evaluating the long-term effects are lacking. It should be noted that asymptomatic or mild SARS-CoV-2 infection in children with cancer, especially haematological malignancies, during intensive chemotherapy in northeast India was associated with a higher risk of death than in cancer patients without infection (Hazarika et al., 2022).

## 4 SARS-CoV-2 infection as a potential risk factor for the development of cancer

It is known that infectious agents participate in oncogenesis, and viral infection is responsible for the development of about 15% of human cancers (Jafarzadeh et al., 2022). There are 7 confirmed viruses involved in the development of cancer: Epstein-Barr virus (EBV), hepatitis B virus (HBV), hepatitis C virus (HCV), human papillomavirus (HPV), human T-lymphotropic virus 1 (HTLV-1), human herpesvirus 8 (HHV-8) and Merkel cell polyomavirus (MCPyV). Oncoviruses can induce epigenetic changes and affect host signal transduction pathways related to cell cycle regulation and metabolism, stimulating inflammation, and promoting angiogenesis, invasion, and metastasis. Moreover, they activate cellular oncoproteins by encoding viral proteins. Furthermore, oncoviruses participate in oncogenesis inducing oxidative stress, affecting genome instability, and increasing the number of mutations (Rapti et al., 2022).

### 4.1 SARS-CoV-2: anti-tumour immunity and immune escape (IE)

The increased incidence or recurrence of cancer in COVID-19 patients may be a consequence of impaired immune surveillance of cancer caused by SARS-CoV-2 infection (Jafarzadeh et al., 2022). CD8^+^ cytotoxic lymphocytes, in acquired immunity kill all types of cancer cells, provided they recognize certain antigens. On the other hand, CD4^+^ lymphocytes play a key role in the antitumor response by inhibiting or stimulating cytotoxic lymphocytes (Ahrends and Borst, 2018). It has been suggested that lymphopenia and a reduced number of CD4^+^ and CD8^+^ T cells are hallmarks of SARS-CoV-2 infection. Moreover, a severe course of SARS-CoV-2 infection is characterised by a reduced number of natural killer cells (NK) and impaired cytokine production by these cells. In addition, overexpression of the inhibitory NK group 2-member A (NKG2A) was observed (O'Connell and Aldhamen, 2020). This mechanism reduced the expression of interferon γ (IFNγ), IL-2, TNF-alpha, CD107a, and granzyme B, as well as the functional depletion of CD8^+^ T and NK cells. Similar changes in the immune system occur in some types of cancer displaying tumour growth (Jafarzadeh et al., 2022). NKG2A is an inhibitory receptor expressed in T and NK cells. Binding NKG2A with CD94, and the Src homology region 2 domain-containing phosphatase-1 (SHP-1), its action interferes with the effector functions of T and NK cells, causing a reduction in anticancer efficacy (André et al., 2018).

Immune escape (IE) is a phenomenon including the mechanisms of immune elimination avoidance. Cancer cells undergo immunoediting, losing their antigenicity or immunogenicity. Moreover, recruiting immunosuppressive leukocytes affects the orchestration of the suppressive microenvironment (Beatty and Gladney, 2015). IE is associated with tumour progression (Gil et al., 2020), metastasis (Schaller and Agudo, 2020), and angiogenesis (Liu and Cao, 2016). Viruses also can enhance IE resulting in an impaired response of the host immune system to the infectious agent. Different mechanisms have been observed among others in HCV, influenza A virus, and SARS-CoV. Several mechanisms affecting immune surveillance have also been reported in SARS-CoV-2 including dysregulation of IFN-I production, cytokines release, dendritic cells, macrophages, NK, and neutrophil cellular function (Chakraborty et al., 2022). However, the relationship between SARS-CoV-2 and cancer immune surveillance is not fully known (Liapis and Baritaki, 2022).

### 4.2 DAMP (damage-associated molecular pattern) and PAMP (pathogen-associated molecular pattern) - possible link between SARS-CoV-2 infection and oncogenesis

Antigenic stimulation induced by DAMP and PAMP molecules in both cancer and infectious diseases seems similar. Once infected by a virus, the immune system uses different mechanisms to recognize and defend against the virus. The first line of host defence with a viral infection, innate immunity, involves responding against the virus by recognizing PAMPs and DAMPs via transmembrane or intracellular pattern recognition receptors (PRRs) (Li and Wu, 2021). NK cells play a pivotal role in defence against SARS-CoV-2 infection. NK cells releasing cytotoxic granules, participating in antibody-dependent cellular cytotoxicity, and producing numerous cytokines and chemokines, can kill virus-infected cells (Ma et al., 2021). Moreover, infected cells can undergo inflammatory cell death and release DAMPs, such as viral nucleic acids and oligomers (Yap et al., 2020). DAMPs and PAMPs cause inflammation related to the production of different cytokines, an increase in reactive oxygen and nitrogen species, tissue damage, and apoptosis (Hotchkiss and Moldawer, 2014). It has also been found that hypoxia and the hypoxic microenvironment created by inflammation provoke oxidative stress and likely malignant transformation (Baghban et al., 2020). Moreover, the hypoxic microenvironment induces the synthesis of lysyl oxidase (LOX), which promotes the invasion and migration of tumour cells (Ye et al., 2020). However, many cellular processes are associated with different stages of cancer progression including initiation, progression, and metastasis (Wang et al., 2017). The tumour microenvironment (TME) consists of different types of residual and infiltrating cells, extracellular matrix, and secreted signals that vary significantly between different cancers (Anderson and Simon, 2020).

Cancer-associated fibroblasts (CAFs) play a specific role in stimulating cancer cell growth by releasing growth factors (Xing et al., 2010) and inhibiting the immune response mediated by natural killer (NK) and T-cell activity (Öhlund et al., 2014). Activation of CAF causes an increase in collagen-1 synthesis, resulting in fibrosis that obstructs blood supply and induces hypoxia (Chauhan et al., 2013). Hypoxia results in the expression of immune-inhibitory molecules, blunting the effect of tumour-killing cells and inducing a macrophage-suppressive phenotype (Pinter and Jain, 2017; Wang et al., 2017).

Of interest, SARS-CoV-2 enters cells through ACE receptors resulting in the downregulation of the ACE enzyme and enhancement of angiotensin type 2-ATR1 axis (Hoffmann et al., 2020) and some TME components, including CAF, also express elements of the renin-angiotensin-aldosterone system (RAAS) and RAAS-modulated action of CAF (Pinter and Jain, 2017). Furthermore, macrophages represent a prominent category of immune cells within the tumour environment, and tumour-associated macrophages (TAMs) can be divided into two distinct phenotypes, namely, M1 and M2. The emergence of the M1 phenotype is prominent in the early stages of oncogenic processes, but as the tumour evolves, exposure to hypoxic conditions within the TME drives a transition to the M2 phenotype. This transition is particularly induced by IL-4, IL-10, IL-13 and macrophage colony-stimulating factor (M-CSF). It appears that cytokine-driven M2 macrophages influence tumour growth dynamics and remodelling of the TME. Furthermore, hypoxia has been suggested to play a key role in modulating tumour immunity. TME hypoxia has a significant impact on the direct transformation of TAM to a functionally M2-like state. This transformation is mediated by mechanisms including metabolic adaptations, lactic acidosis, angiogenic processes, and structural reconfiguration of stromal elements. As a result, M2-like TAMs are induced to actively engage in activities directed towards immunosuppression, angiogenesis, and other supportive processes essential for the maintenance of the tumour milieu (He and Zhang, 2021). Similar patterns of immune response have been observed during SARS-CoV-2 infection. During the progression of COVID-19, there is a notable shift from the Nod-like receptor family, pyrin-containing 3 (NLRP3) cytokine storm to a state of compensatory immunosuppression and transformation of macrophages to the anti-inflammatory M2 phenotype (du Plessis et al., 2022). Moreover, cancer-associated adipocytes (CAA) are a key component that actively participates within the TME. Through these mechanisms, including mutual communication with cancer cells through the exchange of cytokines and lipids, CAA exerts a discernible influence on the acquisition of pro-inflammatory and invasive phenotypes in the latter. CAA-generated IL-6 significantly enhances the invasive potential of neoplastic cells, thereby increasing the spread of metastatic lesions. The bidirectional interaction between tumour cells and peritumoral adipocytes is established. A possible energy source for cancer cells may be the activation of lipolysis by cancer cells and the increased release of free fatty acids (FFA). In addition, increased expression of adipose triglyceride lipase (ATGL) in tumour cells after contact with adipocytes is associated with tumour aggressiveness and invasiveness. ATGL is involved in the lipolytic pathway and affects the release of FFA stored in cancer cells into the microenvironment. In addition, tumour cell-CAA interactions are associated with the production of intracellular reactive oxygen species (ROS) and activation of the HIF1/MMP14 pathway which promotes cancer invasion (Bouche and Quail, 2023). Resistance to oncolytic virus therapy has also been shown to be a lipid-dependent phenomenon (Surendran et al., 2023). It has been suggested that EBV infection modulates gene expression in adipocytes, leading to dysregulation of their functionality and consequent changes in the tumour microenvironment (Liu et al., 2021).

### 4.3 SARS-CoV-2: cytokine storm and oxidative stress

A cytokine storm related to an overactive immune response is a state of a systemic inflammatory syndrome associated with elevated levels of circulating cytokines. This life-threatening condition can be triggered by different factors, including SARS-CoV-2 infection. Increased cytokine levels in COVID-19 including IL-1β, IL-6, interferon-inducible protein 10 (IP-10), TNF-alpha, IFN-γ, macrophage inflammatory protein (MIP) 1α and 1β, and vascular endothelial growth factor (VEGF) were observed (Fajgenbaum and June, 2020). It has been suggested that IL-6 levels are an important predictor of COVID-19 severity (Wang et al., 2020a; Aziz et al., 2020; Chen et al., 2020). IL-6 is one of the factors involved in the induction of inflammation, oncogenesis, and cytokine storm. In addition, IL-6 production by ageing cells is associated with age-dependent pathologies and cancer. The action of IL-6 is exerted mainly by activators of the transcription 3 (STAT3) pathway. Moreover, the involvement of IL-6 in multiple signal transduction pathways regulating survival, cell proliferation, angiogenesis, tumour development and progression by the expression of several genes indicates an important role in cancer (Hirano, 2021). Furthermore, higher levels of TNF-alpha were observed in patients with severe/critical than mild or moderate courses of COVID-19 (Balkwill, 2006). Similarly, the overexpression of TNF-alpha was found in numerous cancers including ovarian, breast, and colorectal (Kulbe et al., 2007; Al Obeed et al., 2014; Liu et al., 2020). This cytokine participates in chronic inflammation, apoptosis, angiogenesis, and immunity (Kobelt et al., 2020). TNF-alpha acting by TNFR1 receptors inhibits cancer development while activating TNFR2 promotes cancer development. Moreover, TNF-alpha altering the microenvironment increased tumour invasiveness and promoted cancer metastasis (Ham et al., 2016). Furthermore, elevated levels of chemokines that contribute to cancer development, such as CCL2, CCL4, CXCL8, CXCL9 and CXCL10 have been found in COVID-19. These chemokines participated in oncogenesis promoting tumour cell expansion, cancer stem cell proliferation, metastasis, angiogenesis, induction of epithelial-mesenchymal transition, the attraction of myeloid-derived suppressor cells and recruitment of fibroblast (Jafarzadeh et al., 2022).

In addition, SARS-CoV-2 infection-dependent deprivation of ACE2 receptors on the cell surface and increased pro-inflammatory and oxidative effects of angiotensin II promote oxidative stress. The production of ROS may also be associated with macrophage activity in acute COVID-19 and treatment with mechanical ventilation (Alpalhão et al., 2020). ROS have been involved in cancer development by several mechanisms, including oxidative damage to cellular macromolecules by impaired antioxidant and/or DNA repair mechanisms, and altered gene expression patterns (Klaunig et al., 2010).

### 4.4 SARS-CoV-2 genome integration and cancer induction

The mechanism of integration of the viral genome into the host genome has been well known, especially for the most important viral carcinogens including HBV, HCV, EBV and HPV (Plummer et al., 2016). Insertion of both DNA and RNA of the viral genome into the DNA of the host cell causes insertional mutagenesis and viral survival in cells. The transformed cell enters an immortal state and acquires unlimited replicative potential (Akram et al., 2017). Epigenetic changes have been linked to oncoviruses-mediated cancer development. Oncoviruses cause host DNA methylation, histone modification, chromatin remodelling and virus-encoded non-coding RNAs (e.g., microRNAs, long non-coding RNAs, circular RNAs) resulting in control of cellular gene expression and changes in the host cell genome (Kellogg et al., 2021; Pietropaolo et al., 2021).

There are some hypotheses describing the integration of SARS-CoV-2 into the human genome (Dai et al., 2020; Zhang et al., 2021). Prolonged detection of SARS-CoV-2 RNA in non-infectious individuals (Li et al., 2020) and recurrence of PCR-positive tests after recovery from COVID-19 with some “re-positive” cases not due to reinfection were observed (Yuan et al., 2020; Yahav et al., 2021).

First, SARS-CoV-2 is a positive-strand RNA virus that, like other beta-coronaviruses, uses RNA-dependent RNA polymerase to replicate its genomic RNA and transcribe subgenomic RNA (Zhang et al., 2021). Moreover, it has been found that viral RNA is reverse-transcribed in human cells by reverse transcriptase (RT) from long interspersed nuclear elements (LINE) (Kazazian and Moran, 2017). SARS-CoV-2 sequences can integrate into the host cell genome through a LINE1-mediated retro position mechanism (Zhang et al., 2021). LINE-1 in human cells was induced to be over-expressed after SARS-CoV-2 infection or after a cytokine storm associated with SARS-CoV-2 *in vitro*. However, other studies showed that retro transposition of the SARS-CoV-2 genome involving LINE-1 into host DNA is rare (Briggs et al., 2021).

### 4.5 SARS-Cov-2 and stimulating signalling in oncogenic pathways

#### 4.5.1 IL-6/JAK/STAT signalling pathway

SARS-CoV-2 infection causes activation of certain signalling pathways, including Janus kinase/signal transducer and activator of transcription (JAK/STAT), nuclear factor kappa B (NFκB), interferon response factor (IRF) 3 and 7. The production of pro-inflammatory cytokines, described above, in infected cells is increased by this signalling cascade (Rahimmanesh et al., 2022). IL-6 participates in oncogenesis and anti-apoptosis signalling (Vargas and Harris, 2016). IL-6 activates both traditional and trans-signalling pathways of JAK-STAT3 signalling. In cancer cells, IL-6 increases the expression of downstream STAT3 targets (Chang et al., 2013). A key role for IL-6/JAK/STAT3 in the regulation of the growth, survival, invasiveness, metastasis, and progression of many cancers was shown. Moreover, IL-6/JAK/STAT3 inhibits the anti-tumour immune response (Kumari et al., 2016). Hyperactivation of the JAK/STAT pathway can cause the development of different types of cancer and is associated with poor clinical prognosis (Johnson et al., 2018; Braicu et al., 2019). Increased levels of IL-6 in the tumour microenvironment and/or mutations of loss-of-function mutations affecting STAT3 negative regulators result in STAT3 hyperactivation in tumour cells (Walter et al., 2009). In addition, the ability of STAT3 to promote IL-6 gene expression by binding to the IL-6 promoter results in a positive autocrine feedback loop (Hirano and Murakami, 2020). It should be noted that STAT3 promotes angiogenesis, invasiveness, metastasis, and immunosuppression. Thus, activation of the IL-6/JAK/STAT pathway, related to SARS-CoV-2 infection and some cancers, may play an important role in oncogenesis.

#### 4.5.2 Nuclear factor κB pathway

NFκB signalling is involved in inflammation, cellular immunity and stress as well as plays a key role in the synthesis of numerous chemokines and cytokines. This pathway may be activated by viral genetic materials or proteins (de Wit et al., 2016; Ma et al., 2020). Hyperactivation of the NFκB pathway participated in the pathogenesis of severe or critical SARS-CoV-2 infection (Song et al., 2017). One of the key mechanisms of activation of NFκB after coronavirus infection is the MyD88 pathway acting by PRRs. This results in the expression of pro-inflammatory cytokines including IL-6, TNF-alpha, and chemokines (Sau et al., 2016). It is suggested that the detection of viral proteins by the innate immune system results in the hyperactivation of NFκB plays a key role in the COVID-19 cytokine storm, extrapulmonary symptoms of SARS-CoV-2 infection and mortality (Kumari et al., 2016). Hyperactivation of NFκB also participates in oncogenesis. In addition, the NFκB pathway is a key target in the treatment of different types of cancer (Sau et al., 2016; Ma et al., 2020).

#### 4.5.3 Type I interferon (INF-I) signalling

Interferons (IFNs) are members of a large family of cytokines that are currently classified based on receptor specificity and sequence homology into three groups (type I, II, and III IFNs).

INF-I, as IL-6 binds to the JAK-activating receptor and initiates signal transduction by the JAK/STAT pathway resulting in the activation of multiple interferon regulatory factors (IRFs) and IFN-stimulated genes (ISGs), that promote inflammatory and innate antiviral response (Snell et al., 2017). It has also been shown that IFN-I plays a key role in inhibiting tumour proliferation and promoting tumour cell senescence and death. While impaired IFN-I signalling is associated with tumour progression (Fuertes et al., 2011; Lamsal et al., 2023). In addition, the crucial role of IFN-I response during the early phase of viral infection was found. Similarly, to other viruses, SARS-CoV-2 has evolved mechanisms to evade the host antiviral response. It has been suggested that IFN-I signalling is suppressed in response to SARS-CoV-2 infection (Blanco-Melo et al., 2020). In patients with severe and critical course of COVID-19 and with high blood viral excessive type I IFN response causes activation of NFκB-related inflammatory response associated with increased TNF-alpha and IL-6 synthesis (Hadjadj et al., 2020). However, data describing the IFN-I signalling in SARS-CoV-2 infection are inconclusive. Some studies found intensified IFN-I response and expression of multiple IFN-stimulated genes in bronchoalveolar lavage fluid (Wilk et al., 2020; Zhou et al., 2020). Moreover, IFN-I response co-existed with the TNF-alpha/IL-1β-driven inflammation was observed in patients with severe COVID-19 (Lee et al., 2020; Lucas et al., 2020). While, an excessive but delayed IFN-I immune response was shown in mouse models during SARS-CoV-2 infection, associated with increased infiltration and recruitment of monocytes and macrophages into infected lungs and depleted T-cell responses resulting in fatal pneumonia (Channappanavar et al., 2016). Thus, impaired IFN-I signalling induced by SARS-CoV-2 infection may cause an ineffective anti-tumour response and tumour progression.

### 4.6 SARS-CoV-2 and cell cycle dysregulation

It has been suggested that the main oncogenic effect of SARS-CoV-2 infection is cell cycle dysregulation. Non-structural proteins 3 (Nsp3) and 15 (Nsp15) of SARS-CoV-2 cause the degradation of tumour suppressor proteins P53 and retinoblastoma (Rb), respectively (Bhardwaj et al., 2012; Ma-Lauer et al., 2016). Furthermore, it has been shown that the S2 subunit of SARS-CoV-2 interacts strongly with the tumour suppressor P53 and BReast CAncer gene 1/2 (BRCA 1/2) (Singh and Bharara Singh, 2020). Furthermore, the ring-finger ligand of the cellular E3 ubiquitin ligase and zinc-finger domain of CHY1 (RCHY1) are the interaction partners of the viral SARS-unique domain (SUD) and papain-like protease (PLpro); the result engages cellular p53 as an antagonist of coronavirus replication. Human coronaviruses antagonise the viral p53 inhibitor by stabilising RCHY1 and promoting RCHY1-mediated p53 degradation (Policard et al., 2021). Another possible mechanism promoting malignancies by SARS-CoV-2 is altering the activity of the transcription factors E2F RB1. The transition from the G1 to the S phase in the cell cycle is controlled by Rb by modulation of E2F activity. It has also been shown that RB1 activity was significantly decreased and E2F increased in patients with COVID-19. This suggested that SARS-CoV-2 inactivates the tumour suppressor Rb resulting in elevated E2F activity and promoting cell proliferation like some other oncogenic viruses (Rahman et al., 2021).

Another possible mechanism responsible for the development of cancer after SARS-CoV-2 infection may be the disruption of the physiological process of apoptosis (Chaudhry et al., 2022). The N protein, the core element of SARS-CoV-2, induces the immunological effect and antibody production in the infected host (Wu et al., 2023) and has a functional domain for RNA binding and viral replication (Bai et al., 2021). The N protein inhibits cell apoptosis by affecting an anti-apoptotic protein, myeloid cell leukaemia-1 (MCL-1) protein (Akgul, 2009), and Casp-3 cleavage (Elmore, 2007). Thus, the SARS-CoV-2 component N protein may be one of the factors involved in the oncogenic effect of SARS-CoV-2 infection (Pan et al., 2023).

**FIGURE 1:**
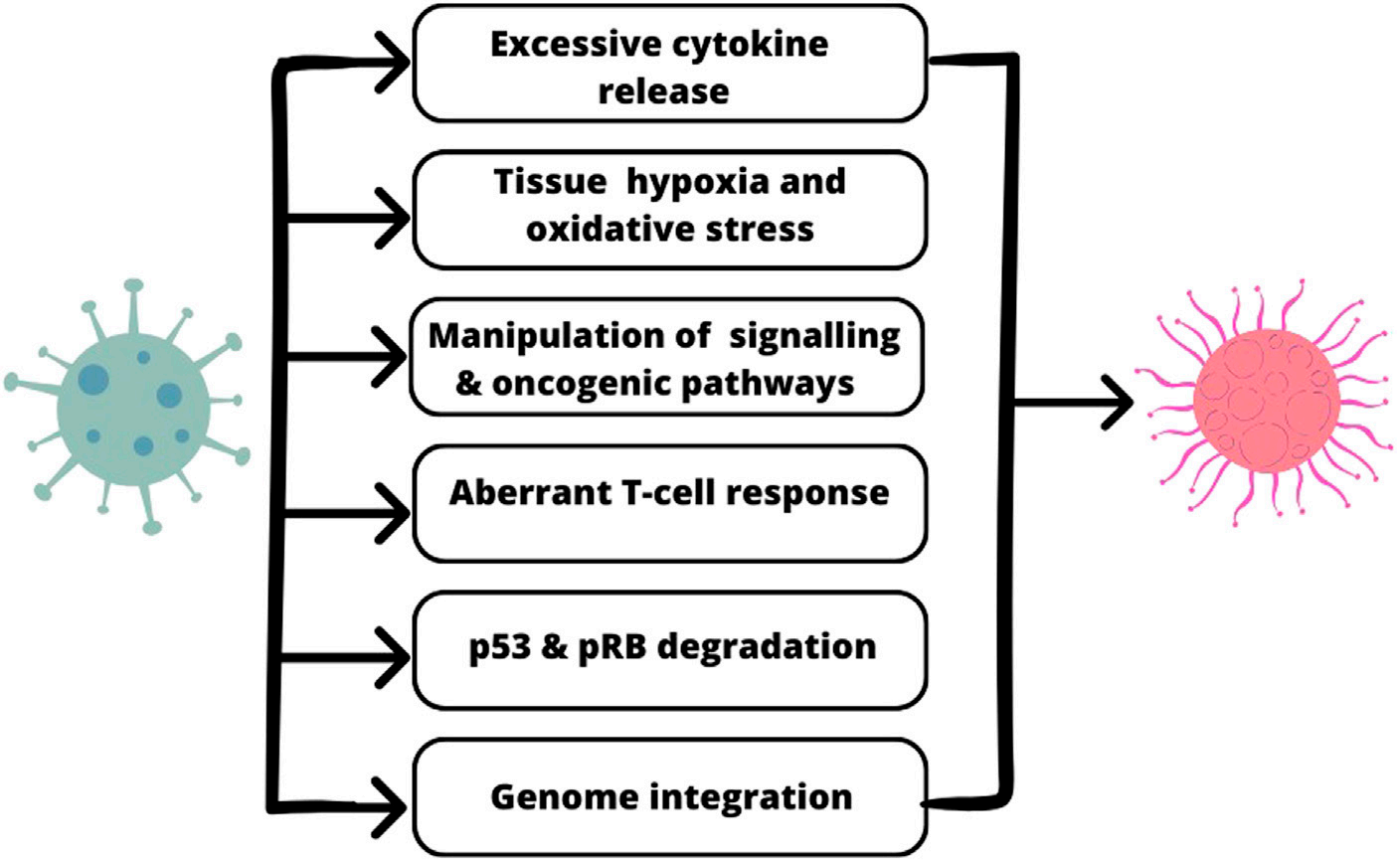
The shared molecular mechanisms between SARS-CoV-2 oncogenesis.

The association between molecular mechanisms of SARS-CoV-2 action and oncogenesis is presented in Figure 1.

### 4.7 The impact of SARS-CoV-2 on renin-angiotensin system (RAS) function and the development of haematological malignancies

The disturbances of COVID-19 infection, among others, may include lymphopenia, thrombocytopenia, and elevated D-dimer levels (Costa et al., 2022). It has also been suggested that SARS-CoV-2 infection influences the development of haematological malignancies (Haznedaroglu and Malkan, 2016). The RAS occurring in the bone marrow microenvironment regulates blood cell production by autocrine, paracrine, and intracrine pathways. Moreover, RAS regulates the proliferation, differentiation, and engraftment of hematopoietic stem cells. Furthermore, angiotensin II type 1a receptors localised on CD34^+^ hematopoietic cells increase the production of hematopoietic progenitors in the bone marrow and cord blood and thus has been suggested that abnormalities in local RAS function in the hematopoietic system are involved in the pathogenesis of leukaemias and other haematological malignancies (Beyazit et al., 2007). Overactive ACE may cause the accumulation of blasts in the bone marrow and their migration into circulation. Moreover, elevated ACE levels decreased N-acetyl-seryl-aspartyl-lysyl-proline tetrapeptide (AcSDKP) levels and impaired the antiproliferative effect of goralatide on hematopoietic cells and blasts. Angiotensin may also be an autocrine growth factor for acute myeloid leukaemia cells (Wiese et al., 2020). SARS-CoV-2 enters cells by ACE2 and downregulates ACE2 expression and suppresses the protective RAS pathway (Nekooghadam et al., 2021). It may be a link between SARS-CoV-2 infection and the development of haematological malignancies in predisposed individuals. For example, the case of a 35-year-old man without comorbidities diagnosed with acute lymphoblastic leukaemia 2 months after SARS-CoV-2 infection was described. A short period between SARS-CoV-2 infection and the development of hematologic malignancy has also been observed in a 36-year-old man diagnosed with myelodysplastic syndrome with excess type 1 blast and in a 31-year-old woman with acute myeloid leukaemia (AML) (Haznedaroglu and Malkan, 2016). Moreover, significant deterioration of laboratory tests and the onset of AML was reported in a patient with a severe, complicated course of COVID-19 (Ioannidou et al., 2021). The association between SARS-CoV-2 and acute leukaemia in children has also been suggested. According to Graeves’ hypothesis, the virus acting as a second triggering step may stimulate an exaggerated immune response in the pre-leukemic clonal population, causing the proliferation of leukemic cells (Chen et al., 2022).

### 4.8 SARS-CoV-2 and epigenetic changes and genetic mutations

Epigenetic modifications include changes in gene expression without changes in the underlying DNA sequence. DNA methylation and histone modifications alter DNA accessibility and chromatin structure, resulting in gene expression patterns (Handy et al., 2011). Moreover, transient changes in chromatin structure and post-transcriptional modifications by non-coding RNAs (ncRNAs) result in epigenetic modifications (Mattick et al., 2023). Such modifications influence an individual’s susceptibility to infection and may be involved in the development of cancer. SARS-CoV-2 infection is associated with the methylation of the ACE2 receptor (Pinto et al., 2020). Moreover, SARS-CoV-2 viral proteins interact with several host epigenetic enzymes including histone deacetylases (HDACs) and bromodomain-containing proteins, resulting in antagonism of cell signalling (Behura et al., 2023). Furthermore, epigenetic factors upregulated by SARS-CoV-2, including PRMT1, TRIM16, HDAC7, HDGF, DTX3L, and downregulated factors, including PRDM1 and PPARGC1A, PADI3, FOXO1 and HELLS have been described (Khan and Islam, 2021). In addition, several host miRNAs target the SARS-CoV-2 genome. A high potential interaction of miR-1307-3p with the 3′UTR of the SARS-CoV-2 genome was found. This miRNA may also control the expression of genes involved in cell survival and proliferation (BCL2, PI3K/Akt pathway), and cellular trafficking (AP2, PIP5K) associated with viral cell entry and spread (Balmeh et al., 2020). Moreover, dysregulation of several miRNAs triggered by SARS-CoV-2 infection and their association with suppression of TLRs, TRAF6 and IFN signalling have been observed (Khan et al., 2020). Thus, it appears that SARS-CoV-2 infection is involved in the development of cancer through epigenetic mechanisms leading to a dysfunctional immune response. However, further studies are needed to explain the epigenetic changes associated with SARS-CoV-2 infection and their links to carcinogenesis.

### 4.9 SARS-CoV-2 and oncolytic effects

It is known that some viruses, natural or genetically modified, can kill cancer cells by infecting them and intensively replicating, leading to lysis (Mondal et al., 2020). Moreover, antiviral immune responses associated with oncolytic virus infection can prevent the immune escape of cancer cells (Wang et al., 2020b). Experimental studies showed cytopathogenic effects of SARS-CoV-2 delta virus infection on clear cell and papillary renal cell carcinoma (Choong et al., 2023). Another study found SARS-CoV-2 to be an oncolytic virus in acute leukaemia (Kandeel et al., 2021). Furthermore, tumour regression was observed in colorectal cancer during acute infection with SARS-CoV-2 (Ottaiano et al., 2021). The proposed mechanism includes a direct oncolytic effect (Ottaiano et al., 2021) and an enhanced immune response against virus-infected cancer cells (Brown et al., 2017). Of interest, colon cancer cells express ACE receptor and neurolipin (NRP-1), which facilitate their infection by SARS-CoV-2 resulting in direct cytotoxic T cell immune action (Daly et al., 2020). In addition, the massive pro-inflammatory cytokine release induced by SARS-CoV-2 infection, including IL-2, IL-6 and TNF-alpha attracts NK and T cells to the neoplastic cells and enhances the immune response (Pasin et al., 2020).

### 4.10 Reactivation of dormant cancer cells (DCC)

SARS-CoV-2 infection can activate dormant cells that survive cancer treatment, which is associated with reduced numbers and activity of NK and T cells and altered activation of monocytes, macrophages, and neutrophils (Anderson and Simon, 2020). One of the lines of defence against pathogens is the neutrophil-derived network-like structure of DNA strands and proteins called the extracellular neutrophil trap (NET) (Barnes et al., 2020; Francescangeli et al., 2020). The NET releases high concentrations of anti-pathogen factors, creating a physical barrier to pathogens. Recent data suggest that neutrophils and NETs play an important role in the stimulation of dormant cancer cells (DCC) (Francescangeli et al., 2020). It has been suggested that the activity of proteases, elastases, and metalloprotease 9 (MMP-9) results in the degradation of laminin, revealing the formation of new epitopes that stimulate cell proliferation and metastasis. The cytokine storm and NET perturbations associated with SARS-CoV-2 infection may be responsible for the stimulation of DCC. The main mechanism appears to be the activation of the NF-kappa pathway by high levels of IL-6, acting directly by increasing cell proliferation or indirectly by creating an environment conducive to the metastatic process (De Cock et al., 2016). It should also be noted that the prolonged immune response to SARS-CoV-2 infection is a cause of immune system exhaustion. It favours the activation of DCC (Qin et al., 2020).

### 4.11 The disruption of the tumorigenic environment, immune inhibition, surveillance, and immunosuppression

SARS-CoV-2 infection with immune system suppression and immunosuppression creates an optimal tumourigenic environment for pre-malignant, malignant, and dormant cells. Similarly, inflammatory infiltrates and high levels of cytokine expression in the tumour microenvironment have been reported, particularly in the later stages of the disease (Grivennikov and Karin, 2010; Grivennikov and Karin, 2011). As mentioned above, a hyper-stimulated immune response and a subsequent cytokine storm have been observed in SARS-CoV-2 infection. This results in a feedback loop that cannot be mediated by anti-inflammatory factors, and subsequent systemic inflammation in which TNF-alpha and IL-6 play a key role in cancer progression (Balkwill, 2009; Grivennikov et al., 2010). Moreover, activation of the NLRP3 inflammasome plays an important role in promoting tumour growth and metastasis in both SARS-CoV-2 infection and some cancers, including breast, colon, lung, and cervical cancers (He et al., 2006; Guo et al., 2016; Moossavi et al., 2018; Freeman and Swartz, 2020). Another factor associated with SARS-CoV-2 infection that favours the progression of tumourigenesis is lymphocyte functional exhaustion, which causes gradual attrition of effector functions within T-cell populations, accompanied by metabolic perturbations, impaired memory retrieval, disrupted homeostatic self-renewal and changes in epigenetic programming. These changes are associated with increased activation of these lymphocytes by elevated levels of IFN-γ and TNF-alpha. The depletion of functional capacity within T lymphocytes impairs their ability to effectively inhibit tumour progression (du Plessis et al., 2022; Baitsch et al., 2011; Watowich et al., 2023; Saka et al., 2020; Slaney et al., 2013; Chen et al., 2021).

## 5 Potential impact of antiviral therapy on cancer

Some approved drugs are currently available for the treatment of COVID-19 including remdesivir and molnupiravir. Several of them may increase the risk of cancer. It has been shown that remdesivir, but not monupiravir, induced lytic reactivation of Kaposi’s sarcoma-associated herpesvirus (KSHV), and EBV, the two major oncogenic herpesviruses, in one patient (Chen et al., 2022). Furthermore, KSHV + patients, especially in endemic areas exposed to SARS-CoV-2 or undergoing treatment, may increase the risk of the development of virus-related cancers, even after fully recovering from COVID-19 (Chen et al., 2021). On the other hand, the spontaneous immunological reaction to SARS-CoV-2 infection may induce an anti-tumour response (Challenor and Tucker, 2021). Alleviation of lymphadenopathy in a patient with classical Hodgkin’s lymphoma (EBV-positive) after infection with SARS-CoV-2 has been reported (Challenor and Tucker, 2021).

Glucocorticoid therapy is used as one of the therapeutic options in the severe course of COVID-19 (Garassino et al., 2020; Sahu et al., 2021). It has been shown that the risk of death in patients receiving oxygen therapy with and without invasive mechanical ventilation was reduced by 35% and 20%, respectively after dexamethasone treatment (Horby et al., 2021). In addition, treatment with glucocorticoids in the severe course of COVID-19 decreased IL-6 levels (Xiang et al., 2020). IL-6 is a tumorigenic driver, an anti-apoptotic signal, and a pivotal biomarker in cancer diagnosis and prognosis (Ryan et al., 2014). Therefore, it would be crucial to assess not only the protective effect of IL-6 inhibitors on inflammation caused by COVID-19 but also their therapeutic implications in cancer therapy (Turnquist et al., 2020).

## 6 Long-COVID and potential long term effects of SARS-CoV-2 infection

Long-COVID is a term first used in social media to describe the occurrence of symptoms associated with SARS-CoV-2 infection, regardless of the viral status (Raveendran et al., 2021). Currently, it is estimated that 65 million people worldwide have long-COVID (Ballering et al., 2022). In people with a long COVID, the occurrence of one or more symptoms of acute COVID-19 or the presence of new symptoms was observed. In addition, long-COVID may be continuous or recurrent and reversible. The most frequent symptoms of long-COVID include fatigue, reduced quality of life, shortness of breath, arthralgia, and chest pain (Ballering et al., 2022). The impact of long-COVID on the course and outcome of cancer patients has been demonstrated (Cortellini et al., 2022; Monroy-Iglesias et al., 2022; Dagher et al., 2023). Long-COVID was associated with elevated levels of pro-inflammatory cytokines and an impaired T-cell response that persisted several months after the infection was resolved (Hempel et al., 2020). Studies assessing the oncogenic effects of long-COVID are necessary. The possible development of cancer and acceleration of cancer progression associated with SARS-CoV-2’s ability to modulate oncogenic pathways, promote low-grade chronic inflammation, and cause tissue damage should prompt thoughtful, long-term clinical trials (Saini and Aneja, 2021).

Another potential oncogenic effect of SARS-CoV-2 infection may be associated with the development of chronic conditions related to an increased risk of cancer. Increased prevalence of obesity in children and adolescents (Anderson et al., 2023) and obesity, pre-diabetes and diabetes in adults (Stiegmann et al., 2023) during the COVID-19 pandemic were shown. It has been suggested that COVID-19-induced diabetes is a novel form associated with beta-cell damage and insulin resistance caused by SARS-CoV-2 infection (Joshi and Pozzilli, 2022). Multiple abnormalities, including glucose and lipid metabolism, abnormal cytokine and adipokine profiles, and enhancement of insulin/IGF-1 signalling are associated with the development of cancer-related to obesity (Scully et al., 2021).

## 7 Conclusion

Both the SARS-CoV-2 pandemic and its aftermath pose extreme challenges to health systems. Recent studies suggest pathogenetic mechanisms common for both SARS-CoV-2 and oncogenesis. SARS-CoV-2 exploits host immunity stimulates signalling and oncogenic pathways and may establish an oncogenic microenvironment. Persons with clinically recovered COVID-19 show profound immune alterations that persist for several months after hospital discharge. Patients with cancer are at higher risk of SARS-CoV-2 infection, severe clinical illness, cancer progression and death. Therefore, this group of patients requires special care in terms of adequate prevention of viral transmission and monitoring of the course of the primary disease. Further studies are needed to determine the long-term impact of asymptomatic or mild symptoms of SARS-CoV-2 infection on the course of the primary disease in cancer patients. Moreover, all patients should be regularly screened for cancer after SARS-CoV-2 infection, as the virus has been shown not only to affect cancer progression but also to induce oncogenesis and cancer recurrence. It should also be noted that cases of a beneficial effect of SARS-CoV-2 infection on the course of the neoplastic process have been described. It is therefore necessary to carry out both experimental and clinical studies that will resolve the existing doubts in the long term.
